# *IL18* Polymorphism and Periodontitis Susceptibility, Regardless of *IL12B*, *MMP9*, and Smoking Habits

**DOI:** 10.1155/2019/9585964

**Published:** 2019-04-01

**Authors:** Patrícia Yumeko Tsuneto, Victor Hugo de Souza, Josiane Bazzo de Alencar, Joana Maira Valentini Zacarias, Cléverson O. Silva, Jeane Eliete Laguila Visentainer, Ana Maria Sell

**Affiliations:** ^1^Post Graduation Program in Biosciences and Physiopathology, Department of Clinical Analysis and Biomedicine, Maringá State University, Paraná, Brazil; ^2^Department of Dentistry, Maringá State University, Paraná, Brazil; ^3^Post Graduation Program in Biosciences and Physiopathology, Basic Health Sciences Department, Maringá State University, Paraná, Brazil

## Abstract

Genetic variations contribute to the susceptibility in the development of periodontitis. The aim of this study was to investigate the influence of *IL18*, *IL12*, and *MMP9* polymorphisms in the chronic periodontitis. This case-control study involved 381 individuals matched by gender and age. Genotyping of *IL18* (rs187238 and rs1946518) and *IL12B* (rs3212227) was performed by PCR-SSP and PCR-RFLP was used for *MMP9* (rs3918242). IL-18 and MMP-9 were quantified in the serum by ELISA. SNPStats and OpenEpi software were used for statistical analysis and, in order to eliminate smoking as a confounding factor, the analyses were also performed in nonsmoking subjects. The *IL18*-137G/C genotype was associated with the risk of chronic periodontitis in nonsmokers (*P*_c_ = 0.03; OR = 1.99; overdominant inherence model). In the multivariate analyses, homozygous *IL18*-137G/G and *IL18*-607C/C were more frequent in males compared to women with these same genotypes (OR = 2.51 and OR = 3.30, respectively). The serum levels of the IL-18 in patients were higher than those in healthy controls (*P* = 0.005). *IL12B* and *MMP9* polymorphisms and MMP-9 serum concentration were similar in patients and controls. In this study, *IL18* was associated with chronic periodontitis susceptibility. Men had greater risk than women for developing the disease when *IL18* polymorphism was considered and the susceptibility was independent of the smoking status.

## 1. Introduction

Chronic periodontitis (CP) is a complex and common oral disease of microbial origin, characterized by inflammatory responses that affect the supporting tissue of the tooth, resulting in the formation of a periodontal pocket and alveolar bone resorption [1, 2]. Eventually, it leads to tooth loss in adult humans impacting their quality of life [3]. Despite the presence of bacteria, immune response is involved in the pathogenesis of CP and genetic polymorphisms in the mediators of immunity have been associated with the susceptibility and severity of periodontitis [4–6]. Among the immune mediators, interleukins (ILs) and matrix metalloproteinases (MMPs) are related to the development of inflammatory response, remodeling of periodontal tissue and bone resorption [7].

IL-18 is a proinflammatory cytokine that stimulates the migration of neutrophils, amplifies IFN-*γ* secretion through NK cells, and activates osteoclast [8]. Studies have indicated that IL-18 induces the release of matrix metalloproteinase 9 (MMP-9) and IL-1*β*, both with proinflammatory activity, resulting in tissue degradation [9]. The IL-18, belonging to the IL-1 cytokine superfamily, is involved in a wide variety of inflammatory diseases [10–12]. It is mainly produced by monocytes, active macrophages, and dendritic cells in response to antigenic stimuli such as lipopolysaccharide of Gram-negative bacteria [13].

The interleukin-12 (IL-12) is expressed through activated macrophages and acts on T and NK cells [14]. This is an immunoregulatory cytokine which affects the Th1 response contributing to the production of interferon gamma (IFN-*γ*) and tumor necrosis factor alpha (TNF-*α*) inducing bone and cartilage resorption by osteoclasts. IL-12 also positively regulates the expression of IL-18 functional receptor and synergizes with IL-18 to improve the production of IFN-*γ* and IL-1*β* [15].

MMPs are a structural and functional family of proteolytic enzymes responsible for the degradation of collagen fibers and extracellular matrix components. These enzymes are produced mainly by polymorphonuclear leukocytes, keratinocytes, monocytes, fibroblasts, and mesenchymal cells. MMPs may play an important role in tissue remodeling and repair associated with the development of inflammatory response [16].

**Figure 1:**
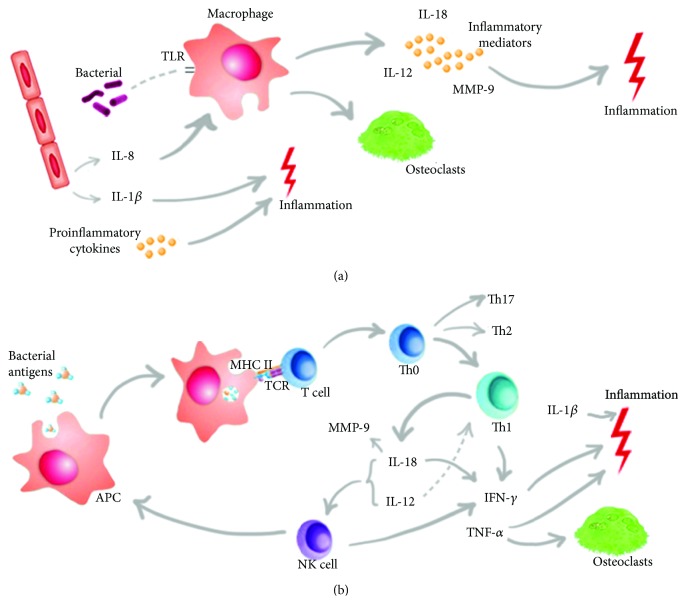
Representation of the biological mechanisms of the cytokines and MMP-9 in the immunopathogenesis of CP. (a) The initial trigger for the immune response is the recognition of components of periodontopathogens, as LPS, by TLRs (Toll-like receptor). This recognition generates an intracellular signaling cascade leading to increased secretion of proinflammatory cytokines, MMPs, and recruitment of osteoclasts by macrophages. (b) This innate immune mechanism of defense may not be sufficient to eliminate the pathogen and with this the adaptive immune response is activated. APCs (antigen-presenting cells) internalize and process bacterial antigens, which bind to MHC II and is transported to the cell surface to be recognized by specific T cell. The Th1 immune response is the main response activated. IL-18 is expressed by macrophages, osteoblasts, fibroblasts, and Kupffer cells, being the main cytokine inducing IFN-*γ*. This cytokine acts synergistically with IL-12 in NK cells to induce IFN-*γ* production and activation of macrophages and dendritic cells that direct the Th1 response. TNF-*α*, IL-1*β*, and proinflammatory cytokines, such as IL-12 and IL-18, orchestrate enzyme-producing events such as MMP-9 and recruitment of osteoclasts, macrophages, and NK cells, causing greater inflammation and destruction of periodontal tissues.

The biological mechanisms involved in CP pathogenesis mediated by IL-18, IL-12, and MMP-9 are described in Figure 1.

Polymorphism in the *IL12B*, *IL18*, and *MMP9* genes could affect the transcriptional activity, the production of proteins, and their serum and crevicular fluid levels in the CP. Therefore, we hypothesize that there was an association between the polymorphisms in the *IL12B*, *IL18*, and *MMP9* regions and the development of periodontitis. Thus, the aim of this study was to investigate the influence of *IL18* (rs187238 and rs1946518), *IL12B* (rs3212227), and *MMP9* (rs3918242) polymorphisms and the IL-18 and MMP-9 serum levels on the immunopathogenesis of CP in individuals from the North/Northwest regions of the state of Paraná, Southern Brazil.

## 2. Material and Methods

### 2.1. Sample Selection

This case-control study was approved by the Human Research and Ethics Committee of the State University of Maringá (COPEP–UEM–number: 719/2011 and 1.866.509/2016). In total, 381 individuals were recruited from the dental clinics of the State University of Maringá (UEM) and Inga University Center (UNINGA) from January 2012 to August 2017. All individuals who agreed to participate in this research were informed about the nature of the study and signed an informed consent form. Clinical parameters of probing depth (PD) and clinical attachment level (CAL) were examined at four sites (mesial, buccal, distal, and lingual) of each tooth, as well as bleeding on probing (BOP), which were realized by the dentist responsible for the clinic, based on the classification of the 1999 workshop [1]. The participants were divided into two different groups: (i) the chronic periodontitis group (CP) was composed by individuals who had at least 5 sites in different teeth with PD ≥ 5 mm, CAL ≥ 3 mm, and more than 25% of BOP; and (ii) the control group was formed by individuals who displayed a PD of less than 4 mm and exhibited less than 25% of BOP. CP patients were classified for their type of extension (localized and generalized) and degree of severity (light, moderate, and severe). According to the classification of periodontal diseases of 2017 [17], the patients in this study can be included in the following categories: stages II, III, and IV based on severity, complexity, extension, and distribution; and grades B (moderate rate of progression) and C (rapid rate of progression). All individuals were from the North and Northwest regions of the state of Paraná (22°29′30^″^-26°42′59^″^S and 48°02′24^″^-54°37′38^″^W), Southern Brazil, and were defined as mixed ethnicity with predominantly European origin [18, 19]. Due to the great Brazilian miscegenation, individuals were classified as previously described by Probst et al. [18] who provided a better picture of Paraná's ethnic constitution, and based on this criterion, descendants of Asians were excluded from the sample. The groups were matched by sex and age. The information about smoking habits was obtained by interviewing the individual (anamnesis).

The inclusion criteria had individuals aged between 30 and 65 years and dental arch with at least 20 teeth. For the determination of serum concentrations for IL-18 and MMP-9, patients and controls were not using antibiotic or anti-inflammatory drugs. The noninclusion criteria included individuals with aggressive periodontal disease, acute infection, diabetes, and rheumatic diseases and individuals who had been treated for periodontitis in the last 6 months. The characteristics of patients with CP and controls are shown in Table 1.

### 2.2. Blood Collection and DNA Extraction

From each individual, 10 ml of peripheral blood was collected in two tubes: one tube without anticoagulant to measure the serum level of cytokines and the other with EDTA for genotyping. DNA was extracted from peripheral blood collected in EDTA using the salting-out method [20]. The concentration and quality of the DNA were analyzed by optical density in Thermo Scientific Nanodrop 2000 apparatus® (Wilmington, USA).

### 2.3. Genotyping of *IL18*

*IL18*-137G>C (rs187238) and *IL18*-607A>C (rs1946518) genotyping was performed by the PCR-SSP according to previous standardization [11]. The DNA concentration used was 50 to 100 ng. For the *IL18*-137 position, we used 0.5 *μ*M of a specific primer sequence and a common reverse primer, 0.3 *μ*M of control primer, MgCl_2_ 1.5 mM, dNTPs 200 *μ*M, and 1.0 U of *Taq* DNA polymerase. For the *IL18*-607 position, we used 0.4 *μ*M of a common reverse primer and 0.4 *μ*M of specific forward primer sequences; in addition, we used 0.13 *μ*M of forward control primer, MgCl_2_ 1.5 mM, dNTPs 200 *μ*M, and 1.0 U of *Taq* DNA polymerase. Amplification cycles were used in a GeneAmp PCR System 9700 (Applied Biosystems™) thermocycler. Amplification product analysis was done on SYBR Safe stained agarose gel (Invitrogen®, Life Technologies, Grand Island, NY).

### 2.4. Genotyping of *IL12B*

*IL12*+1188A>C (rs3212227) genotyping was performed by PCR-SSP using specific genotyping kits (Invitrogen®, Carlsbad, CA, USA) according to the manufacturer's specifications. Visualization of fragment size was performed on 3% agarose gel electrophoresis stained with SYBR Safe DNA Gel Stain (Invitrogen®, Life Technologies, Grand Island, NY).

### 2.5. Genotyping of *MMP9*

*MMP9-*1562C>T (rs3918242) genotyping was performed using PCR-RFLP technique according to Nelissen et al. [21]. For the reaction, 100 ng of DNA, 5 pmol of each primer forward and reverse, 200 *μ*M of each nucleotide, 25 mM MgCl_2_ buffer (PCR Amplification Buffer, Promega), and 0.5 U of *Taq* DNA polymerase were used. Amplification cycles were performed on a GeneAmp PCR System 9700 (Applied Biosystems ™) thermocycler. The amplification products were digested with 5.0 U of the *Sph*I enzyme (Fermentas® Life Science) for 3 hours at 37°C. Analysis of the fragments was performed by electrophoresis on a SYBR Safe stained agarose gel (Invitrogen® Life Technologies, Grand Island, NY).

### 2.6. Determination of the IL-18 and MMP-9 Serum Concentration

Eighteen nonsmoker patients and six nonsmoker controls were selected for IL-18 and MMP-9 serum concentration assays. The CP patients were classified according to disease extension form and on degree of severity (light, moderate, and severe).

The IL-18 and MMP-9 serum concentration was determined using a Human IL-18 ELISA kit (Medical & Biological Laboratories Co. Ltd., code no.7620 Ltd., Nagoya, Aichi, Japan) and Invitrogen Human MMP-9 ELISA kit (Corporation Invitrogen, Catalog #KHC3061, Frederick, Maryland, USA) in accordance with the manufacturer's instructions. Absorbance of each well was read on the ELISA reader (ASYS HITECH GMBH–Eugendorf, Austria) using 450 nm and the reference at 620 nm. Concentrations in the tested sample were estimated using the standard curve. Reactions were done in duplicate.

### 2.7. Statistical Analysis

To evaluate if the estimate genotype distribution between the observed and expected frequencies is found in the Hardy-Weinberg equilibrium, the SNPStats (https://www.snpstats.net/start.htm) statistical program [22] was used. The association between genetic polymorphisms and chronic periodontal disease was assessed using the chi-square test with Yates correction and logistic regression, and the risk was assessed by odds ratio with a 95% confidence interval only for significant *P* value using the SNPstats [22] and OpenEpi Version 2.3 program (https://www.openepi.com/Menu/OE_Menu.htm). Covariate analysis included age, gender, and smoking status. The association tests were realized for codominant, dominant, recessive, overdominant, and log-additive genetic inheritance models where the best inheritance model was defined by the minor Akaike information criteria (AIC) [22]. To eliminate smoking as a confounding factor, the analyses also were done in the nonsmoking patients versus nonsmoking controls. The Bonferroni adjustment for multiple testing was applied and the corrected value (*P*_c_) for a truly significant value was obtained after the multiplication of the *P* value by the number of analyzed SNPs (three SNPs, because of the linkage disequilibrium between *IL18*-137 and *IL18*-607: Δ´ = 0.98, *P* < 10^−16^). The values for cytokine levels were expressed as mean ± SEM (standard error of mean), and for the differences between the groups, the Student *t*-test was used (https://www.graphpad.com/quickcalcs/ttest2/). The Mann-Whitney *U* test was used to analyze the correlation between concentration and genotype (https://www.socscistatistics.com/tests/). All tests were performed at a significance level of 5.0%. Quanto (http://biostats.usc.edu/software) was used to calculate the sample size using the less frequent allele (0.14 for *MMP9*), population risk (50% and OR = 1.9), for statistical power of 80%, and considering the codominant inheritance genetic model.

## 3. Results

The allele and genotype frequency distributions of *IL12B*, *MMP9*, and *IL18* in CP patients and controls are shown in Table 2. The genotype frequency distributions for all genes were consistent with the Hardy-Weinberg equilibrium (*P* > 0.05).

For *IL18*-137, a higher frequency of the G/C genotype was found in patients (50%) when compared to controls (35%) in the nonsmoking group. When analyzing the models of inheritance, significance was observed for codominant and overdominant models, being the overdominant model of choice according to the minor Akaike information criteria (*P*_c_ = 0.03, OR = 1.99, 95% CI = 1.17-3.36) (Table 2). In the analysis of interaction with the covariant gender, *IL18*-137G/G and *IL18*-607C/C genotypes were higher in nonsmoking men (*P*_c_ = 0.03, OR = 2.51, 95% CI = 1.19-5.30 and *P*_c_ = 0.03, OR = 3.30, 95% CI = 1.29-8.40, respectively) when compared with nonsmoking women carrying the same homozygous genotypes (Table 3).

**Figure 2:**
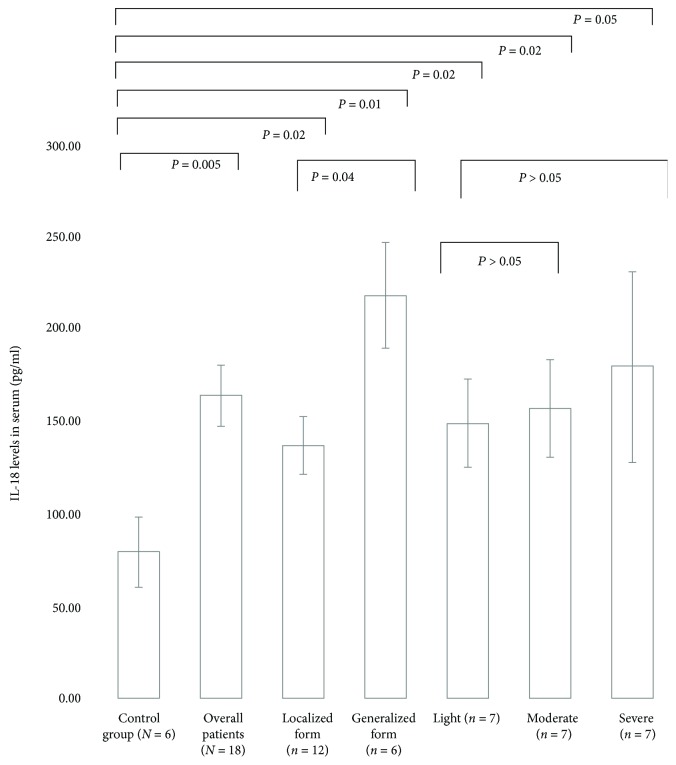
IL-18 serum levels in nonsmoking CP patients (*N* = 18) and controls (*N* = 6). Comparisons were done between the control group and overall patients, CP extension (localized and generalized), and degree of disease severity (light, moderate, and severe); between CP extension (localized *x* generalized) and light degree *x* moderate and severe degree. The results are shown as mean ± SEM. Student's *t*-test was used; *P* ≤ 0.05 was considered significant.

The serum levels for IL-18 in CP patients were higher (164.8 ± 66.4 pg/ml) than those in healthy controls (82.3 ± 43.3 pg/ml; *P* = 0.005). IL-18 concentrations were also higher in different extension forms of CP and in diverse degrees of severity of the disease than those in the controls (Figure 2). The *IL18*-137G/C+G/G and *IL18*-607A/C+C/C genotypes were related to good cytokine production (*P* = 0.012, *Z*-score = 2.50, *U*value = 10 for critical = 17 and *P* = 0.011, *Z*-score = 2.53, *U*value = 8 for critical = 14, respectively).

After analyzing the *IL12B* and *MMP9* polymorphisms, no significant differences in the allele and genotype frequency distributions were observed between CP patients and controls (total sample and nonsmokers). There were no significant differences for the serum levels of MMP-9 between the control group and the several extension forms and severity of CP patients.

## 4. Discussion

In order to evaluate a possible influence of *IL18*, *IL12*, and *MMP9* polymorphisms and IL-18 and MMP-9 serum levels in the immunopathogenesis of the CP, a careful selection of patients and controls was performed in this study. Subjects were matched according to age and gender, and all individuals did not exhibit disease-related disorders, which may influence the course of the disease, such as diabetes, arthritis, and other inflammatory disorders [23–25]. Smoking habits are a risk factor for CP [26, 27]; thus, analyses were also separately done in nonsmoking patients versus nonsmoking controls.

The best results were that *IL18* contributes to the risk for disease independently of the smoking habits and that men had a greater risk than women when these *IL18* polymorphisms were considered. The serum levels of IL-18 were higher in CP patients than in the controls and in patients with more severe and extensive form of the disease.

In this study, the *IL18*-137G/C genotype was found more frequent in the nonsmoking patients and was associated with the risk of CP development. The choice inheritance model was the overdominant that compared the heterozygous genotype versus both homozygous genotypes. This association was observed in nonsmoking individuals and highlights the independency of this confounding factor. The risk was also observed in men carrying the *IL18*-137G/G and the *IL18*-607C/C genotypes when compared with these same genotypes in women. Up to this point, men were considered more susceptive to CP due to the hormonal factors [28] and mainly the personal hygiene habits [29]; however, here it was observed that they had a genotype related to better IL-18 cytokine production which could exacerbate periodontal destruction. *IL18*-137G and *IL18*-607C alleles have been involved in the development of different diseases. The allele *IL18*-607C has been linked to hepatitis C susceptibility [30] and chronic obstructive pulmonary disease [31], and the genotype C/C has been associated with higher IL-18 production in multiple sclerosis [32]; *IL18*-137G was associated with the risk of arthritis [10] and *IL18*-137G/C genotype with oral cancer [12]. More specifically with regard to CP, Li et al. [33] showed in a meta-analysis study, involving nine case-control studies and a total of 576 patients with periodontitis and 458 healthy controls, that the *IL18*-607C and *IL18*-137G alleles were associated with an increased risk of periodontitis. We found no allelic association in our study, possibly due to the small sample of patients.

When the IL-18 serum levels were measured, the IL-18 concentration was statistically significantly higher in CP than in controls and highest concentration was found in the generalized form of the disease. High levels of IL-18 in the serum of the patients with chronic periodontitis were previously documented [33, 34]. Previously study found that *IL18*-137G and *IL18*-607C alleles, located on chromosome 11q22, increase the gene transcription and lead to a higher level of IL-18 protein synthesis [32]. We found that *IL18*-137G/C+G/G and *IL18*-607A/C+C/C genotypes were related to good cytokine production. These facts point to a potential functional role of these variants and their influence on cytokine levels of periodontal tissue and plasma in patients.

*In vivo*, when using IL-18 transgenic (Tg) mice, IL-18 overexpression was related to periodontal disease [35]. When IL-18Tg and wild-type mice were inoculated intraorally with *Porphyromonas gingivalis*, after seventy days of infection, there was periodontal bone loss in IL-18Tg mice but not in wild-type. RT-PCR analysis showed elevated expressions of mRNAs for the receptor activator of nuclear factor kappa-B ligand (a key stimulator for the development and activation of osteoclast) and CD40 ligand (a marker of T cell activation) in the gingiva of IL-18Tg-infected mice [35]. Considering this fact as well as the polymorphisms in IL-18 promoter regions and the increased levels of IL-18 in the plasma of CP patients, which we observed in our and other studies, *IL18* polymorphisms and serum concentration may be useful biomarkers for predicting the development of periodontitis.

IL-18 is the major inducer of IFN-*γ* and acts synergistically with IL-12 on NK cells. IL-18 induces the production of IFN-*γ* that activates macrophages, dendritic cells, and Th1 cells directing immune response to the cellular response. Activated macrophages are a potent cell producing TNF-*α* and other chemical mediators, such prostaglandins-E2, which induce bone and cartilage resorption by activation of the osteoclasts. When in the absence of IL-12, IL-18 induces the immune response of Th2 [36] that could deregulate the specific immune response. This biological mechanism may be responsible for initiation and progression of periodontal tissue destruction, by inducing the genesis of osteoclast and increase secretion of matrix metalloproteinases.

In this studied population, *IL12* and *MMP9* polymorphisms and MMP-9 serum levels were not associated with CP. With regard to IL-12, several studies related the highest level of IL-12 in serum, gingival tissue, and crevicular fluid in periodontal disease [15, 34, 37]. Other investigations including IL-12 determination of serum and crevicular fluid should be done for best conclusions regarding the association of these mediators with CP. As for *MMP9* polymorphism, specifically *MMP9-*1562C>T, a meta-analysis published in 2016 related a reduced risk between the T allele and periodontal disease susceptibility in both Caucasian and Asian populations [38]. Different from our results, Silosi et al. [39] showed that MMP-9 levels in the serum and in the gingival crevicular fluid were significantly higher in CP patients when compared to controls.

The present study had some limitations, such as the nonpairing of the number of individuals in the three studied genes and their polymorphisms. This occurred due to the lack of some samples in the course of the study. The serum level of IL-12 was also not determined.

## 5. Conclusion

IL-18 may confer susceptibility to CP independently of smoking habits and *IL12B* and *MMP9* polymorphisms. Men had a greater risk than women for developing the disease when *IL18* polymorphism was considered. *IL18*-137G>C (rs187238) and *IL18*-607A>C (rs1946518) polymorphisms might influence cytokine levels in the plasma of CP patients.

## Figures and Tables

**Table 1:** Characteristics of patients with CP and controls.

	CP patients	Controls		
	*N* = 192	*N* = 189	*P*	OR (95% CI)
	*n* (%)	*n* (%)		
Gender				
Female	96 (50)	116 (61)		
Male	96 (50)	73 (39)		
Age				
Mean ± sd (year)	47.5 ± 9.1	46.3 ± 8.4		
Smoking				
Smokers+ex-smokers	82 (43)	47 (25)		
Nonsmokers	110 (57)	142 (75)	0.0002	2.25 (1.45-3.49)

Nonsmokers	*N* = 110	*N* = 142		
Gender				
Female	57 (52)	98 (69)		
Male	53 (48)	44 (31)		
Age				
Mean ± sd (year)	47.8 ± 9.2	46.5 ± 8.2		

*n*: number; sd: standard deviation; OR: odds ratio; *P*: *P* value—only significant *P* values are shown; CP: chronic periodontitis.

**Table 2:** Genotype and allele frequency distributions of the *IL12, IL18* and *MMP9* in the CP and nonsmoking CP patients compared to their respective controls.

		All subjects		Nonsmokers					
		CP patients	Controls	CP patients	Controls				
		*n* (%)	*n* (%)	*n* (%)	*n* (%)	*P*	*P*_c_	OR (95% CI)	AIC
*IL18*									
137G>C		*N* = 191	*N* = 185	*N* = 109	*N* = 138				
(rs187238)	G/G	91 (48)	97 (52)	48 (44)	75 (54)				
Genotype	G/C^∗^	85 (44)	70 (38)	54 (50)	48 (35)				
	C/C	15 (8)	18 (10)	7 (6)	15 (11)				
	G/G+C/C^∗^	106 (56)	115 (62)	55 (50)^∗^	90 (65)^∗^	ref			
	G/C^∗^	85 (44)	70 (38)	54 (50)^∗^	48 (35)^∗^	0.01	0.03	1.99 (1.17-3.36)	330.7
Allele	G	267 (70)	264 (71)	150 (69)	198 (72)				
	C	115 (30)	106 (29)	68 (31)	78 (28)				
*IL18*									
607A>C		*N* = 192	*N* = 187	*N* = 110	*N* = 140				
(rs1946518)	A/A	43 (22)	35 (19)	29 (26)	27 (19)				
Genotype	A/C	93 (49)	82 (44)	50 (46)	61 (44)				
	C/C	56 (29)	70 (37)	31 (28)	52 (37)				
Allele	A	179 (47)	152 (41)	108 (49)	115 (41)				
	C	205 (53)	222 (59)	112 (51)	165 (59)				
*IL12B*									
1188A>C		*N* = 128	*N* = 131	*N* = 78	*N* = 114				
(rs3212227)	A/A	63 (49)	66 (51)	40 (51)	59 (52)				
Genotype	A/C	49 (38)	53 (40)	27 (35)	46 (40)				
	C/C	16 (12)	12 (9)	11 (14)	9 (8)				
Allele	A	175 (68)	185 (71)	107 (69)	164 (72)				
	C	81 (32)	77 (29)	49 (31)	64 (28)				
*MMP9*									
1562C>T		*N* = 188	*N* = 185	*N* = 101	*N* = 142				
(rs3918242)	C/C	150 (80)	142 (77)	78 (77)	111 (78)				
Genotype	C/T	37 (20)	40 (21)	23 (23)	29 (20)				
	T/T	1 (1)	3 (2)	0 (0)	2 (2)				
Allele	C	337 (90)	324 (88)	179 (89)	251 (88)				
	T	39 (10)	46 (12)	23 (11)	33 (12)				

*n*: number; ref: reference; OR: odds ratio; *P*: *P* value; CP: chronic periodontitis; AIC: Akaike information criteria. ^∗^*IL18-137*G/C genotype: CP patient vs. control in the nonsmoking group.

**Table 3:** *IL18* genotype frequency distributions between nonsmoker CP and controls considering the interaction analysis with the covariant gender.

	CP patients *n* (%)	Controls *n* (%)	*P*	*P*_c_	OR (95% CI)
*IL18*-137G/G (rs187238)					
Female	22 (48)	51 (68)	ref		
Male	26 (52)	24 (32)	0.01	0.03	2.51 (1.19-5.30)
*IL18*-607C/C (rs1946518)					
Female	14 (45)	38 (73)	ref		
Male	17 (55)	14 (27)	0.01	0.03	3.30 (1.29-8.40)

*n*: number; ref: reference; *P*: *P* value; OR: odds ratio; CI: confidence interval; CP: chronic periodontitis.

## Data Availability

The data used to support the findings of this are included within the article.

## References

[1] Armitage G. C. (1999). Development of a classification system for periodontal diseases and conditions. *Annals of Periodontology*.

[2] Page R. C., Schroeder H. E. (1976). Pathogenesis of inflammatory periodontal disease. A summary of current work. *Laboratory Investigation*.

[3] Meusel D. R. D. Z., Ramacciato J. C., Motta R. H. L., Brito Júnior R. B., Flório F. M. (2015). Impact of the severity of chronic periodontal disease on quality of life. *Journal of Oral Science*.

[4] Baker P. J., Roopenian D. C. (2002). Genetic susceptibility to chronic periodontal disease. *Microbes and Infection*.

[5] da Silva M. K., de Carvalho A. C. G., Alves E. H. P., da Silva F. R. P., Pessoa L. S., Vasconcelos D. F. P. (2017). Genetic factors and the risk of periodontitis development: findings from a systematic review composed of 13 studies of meta-analysis with 71,531 participants. *International Journal of Dentistry*.

[6] Hoçoya L. S., Aparecida M., Jardini N. (2010). Polimorfismo genético associado à doença periodontal na população brasileira: revisão de literatura. *La Revue Odontologique*.

[7] Vokurka J., Klapusová L., Pantuckova P., Kukletova M., Kukla L., Holla L. I. (2009). The association of MMP-9 and IL-18 gene promoter polymorphisms with gingivitis in adolescents. *Archives of Oral Biology*.

[8] de Campos B. O., Fischer R. G., Gustafsson A., da Silva Figueredo C. M. (2012). Effectiveness of non-surgical treatment to reduce IL-18 levels in the gingival crevicular fluid of patients with periodontal disease. *Brazilian Dental Journal*.

[9] Jabłońska E., Izycka A., Wawrusiewicz N. (2002). Effect of IL-18 on IL-1beta and sIL-1RII production by human neutrophils. *Archivum Immunologiae et Therapiae Experimentalis*.

[10] Li L. L., Deng X. F., Li J. P., Ning N., Hou X. L., Chen J. L. (2016). Association of IL-18 polymorphisms with rheumatoid arthritis: a meta-analysis. *Genetics and Molecular Research*.

[11] Liu Y., Lin N., Huang L., Xu Q., Pang G. (2007). Genetic polymorphisms of the interleukin-18 gene and risk of prostate cancer. *DNA and Cell Biology*.

[12] Tsai H.-T., Hsin C. H., Hsieh Y. H. (2013). Impact of interleukin-18 polymorphisms -607A/C and -137G/C on oral cancer occurrence and clinical progression. *PLoS One*.

[13] Foster N., Andreadou K., Jamieson L., Preshaw P. M., Taylor J. J. (2007). VIP inhibits *P. gingivalis* LPS-induced IL-18 and IL-18BPa in monocytes. *Journal of Dental Research*.

[14] Gately M. K., Renzetti L. M., Magram J. (1998). The interleukin-12/interleukin-12-receptor system: role in normal and pathologic immune responses. *Annual Review of Immunology*.

[15] Orozco A., Gemmell E., Bickel M., Seymour G. J. (2006). Interleukin-1 beta, interleukin-12 and interleukin-18 levels in gingival fluid and serum of patients with gingivitis and periodontitis. *Oral Microbiology and Immunology*.

[16] Smith P. C., Muñoz V. C., Collados L., Oyarzún A. D. (2004). In situ detection of matrix metalloproteinase-9 (MMP-9) in gingival epithelium in human periodontal disease. *Journal of Periodontal Research*.

[17] Papapanou P. N., Sanz M., Buduneli N. (2018). Periodontitis: consensus report of workgroup 2 of the 2017 World Workshop on the Classification of Periodontal and Peri-Implant Diseases and Conditions. *Journal of Periodontology*.

[18] Probst C. M., Bompeixe E. P., Pereira N. F. (2000). HLA polymorphism and evaluation of European, African, and Amerindian contribution to the white and mulatto populations from Paraná, Brazil. *Human Biology*.

[19] Parra F. C., Amado R. C., Lambertucci J. R., Rocha J., Antunes C. M., Pena S. D. J. (2003). Color and genomic ancestry in Brazilians. *Proceedings of the National Academy of Sciences of the United States of America*.

[20] John S. W. M., Weitzner G., Rozen R., Scriver C. R. (1991). A rapid procedure for extracting genomic DNA from leukocytes. *Nucleic Acids Research*.

[21] Nelissen I., Vandenbroeck K., Fiten P. (2000). Polymorphism analysis suggests that the gelatinase B gene is not a susceptibility factor for multiple sclerosis. *Journal of Neuroimmunology*.

[22] Solé X., Guinó E., Valls J., Iniesta R., Moreno V. (2006). SNPStats: a web tool for the analysis of association studies. *Bioinformatics*.

[23] Aljehani Y. A. (2014). Risk factors of periodontal disease: review of the literature. *International Journal of Dentistry*.

[24] Alves C., Andion J., Brandão M., Menezes R. (2007). Mecanismos patogênicos da doença periodontal associada ao diabetes melito. *Arquivos Brasileiros de Endocrinologia e Metabologia*.

[25] Bakri I., Douglas C. W. I., Rawlinson A. (2013). The effects of stress on periodontal treatment: a longitudinal investigation using clinical and biological markers. *Journal of Clinical Periodontology*.

[26] Haber J., Wattles J., Crowley M., Mandell R., Joshipura K., Kent R. L. (1993). Evidence for cigarette smoking as a major risk factor for periodontitis. *Journal of Periodontology*.

[27] Khan S., Khalid T., Awan K. H. (2016). Chronic periodontitis and smoking. Prevalence and dose-response relationship. *Saudi Medical Journal*.

[28] Carranza Junior F. A., Newman M. G. (1997). *Periodontia Clínica*.

[29] Machion L., Freitas P. M. d., Cesar Neto J. B., Nogueira Filho G. R., Nociti Jr F. H. (2000). A influência do sexo e da idade na prevalência de bolsas periodontais. *Pesquisa Odontológica Brasileira*.

[30] dos Santos K. N., de Almeida M. K. C., Fecury A. A., da Costa C. A., Martins L. C. (2015). Analysis of polymorphisms in the interleukin 18 gene promotor (-137 G/C and -607 C/A) in patients infected with hepatitis C virus from the Brazilian Amazon. *Arquivos de Gastroenterologia*.

[31] Wang J., Liu X., Xie J., Xu Y. (2013). Association of interleukin-18 promoter polymorphisms with chronic obstructive pulmonary disease in male smokers. *International Journal of Immunogenetics*.

[32] Giedraitis V., He B., Huang W. X., Hillert J. (2001). Cloning and mutation analysis of the human IL-18 promoter: a possible role of polymorphisms in expression regulation. *Journal of Neuroimmunology*.

[33] Li Z. G., Li J. J., Sun C. A., Jin Y., Wu W. W. (2014). Interleukin-18 promoter polymorphisms and plasma levels are associated with increased risk of periodontitis: a meta-analysis. *Inflammation Research*.

[34] Sánchez-Hernández P., Zamora-Perez A., Fuentes-Lerma M., Robles-Gómez C., Mariaud-Schmidt R., Guerrero-Velázquez C. (2011). IL-12 and IL-18 levels in serum and gingival tissue in aggressive and chronic periodontitis. *Oral Diseases*.

[35] Yoshinaka K., Shoji N., Nishioka T. (2014). Increased interleukin-18 in the gingival tissues evokes chronic periodontitis after bacterial infection. *The Tohoku Journal of Experimental Medicine*.

[36] Nakanishi K., Nakanishi K., Yoshimoto T., Tsutsui H., Okamura H. (2001). Interleukin-18 is a unique cytokine that stimulates both Th1 and Th2 responses depending on its cytokine milieu. *Cytokine & Growth Factor Reviews*.

[37] Tsai I. S., Tsai C. C., Ho Y. P., Ho K. Y., Wu Y. M., Hung C. C. (2005). Interleukin-12 and interleukin-16 in periodontal disease. *Cytokine*.

[38] Weng H., Yan Y., Jin Y.-H., Meng X.-Y., Mo Y.-Y., Zeng X.-T. (2016). Matrix metalloproteinase gene polymorphisms and periodontitis susceptibility: a meta-analysis involving 6, 162 individuals. *Scientific Reports*.

[39] Silosi I., Cojocaru M., Foia L. (2015). Significance of circulating and crevicular matrix metalloproteinase-9 in rheumatoid arthritis-chronic periodontitis association. *Journal of Immunology Research*.

